# Regulation of brain iron homeostasis and its influence on cognitive function

**DOI:** 10.1007/s00330-025-12143-6

**Published:** 2026-01-22

**Authors:** Sujung Yoon, Yoonji Joo, Eunji Ha, Suji Lee, Chaewon Suh, Yumi Song, Haejin Hong, Youngeun Shim, Yejin Kim, Hyeonji Lee, Hyeonseok Jeong, Soo Mee Lim, In Kyoon Lyoo

**Affiliations:** 1 Ewha Brain Institute, Ewha Womans University, Seoul, South Korea; 2 Department of Brain and Cognitive Sciences, Ewha Womans University, Seoul, South Korea; 3 Division of Psychology and Cognitive Science, Seoul Women’s University, Seoul, South Korea; 4 Department of Radiology, College of Medicine, Ewha Womans University, Seoul, South Korea; 5 Graduate School of Pharmaceutical Sciences, Ewha Womans University, Seoul, South Korea

**Keywords:** Arterial spin labeling, Brain iron homeostasis, Cerebral blood flow, Cognitive function, Quantitative susceptibility mapping

## Abstract

**Objectives:**

Iron is essential for oxygen transport and neuronal integrity, underscoring the importance of maintaining iron homeostasis for optimal brain function. This study aimed to elucidate the interplay among systemic iron status, brain iron levels, and cerebral blood flow (CBF), with a particular focus on their influence on cognitive performance.

**Materials and methods:**

A total of 332 healthy women without a history of iron-related disorders were recruited and stratified into three groups based on serum iron concentrations: low-iron, reference, and high-iron groups. Brain iron content and CBF were assessed in the basal ganglia (BG) using quantitative susceptibility mapping and arterial spin labeling perfusion-weighted imaging, respectively. Cognitive performance was evaluated using attention-focused assessments.

**Results:**

Although the low-iron group exhibited systemic iron deficiency, BG susceptibility values did not significantly differ from those of the reference group (*p* = 0.13). Path analysis revealed that lower blood iron levels were significantly associated with reduced BG susceptibility (*p* < 0.001), and that both lower blood iron (*p* < 0.001) and reduced susceptibility (*p* = 0.01) were associated with increased BG CBF. Decreased blood iron was associated with impaired attention performance, and a curvilinear relationship was observed between BG susceptibility and attention performance.

**Conclusion:**

These findings indicate a dynamic interaction between systemic and brain iron homeostasis, which influences BG CBF and attention performance.

**Key Points:**

***Question*** *What are the associations among blood iron levels, brain iron content, cerebral blood flow (CBF), and attention performance in healthy women?*

***Findings*** *Reduced systemic and brain iron levels were significantly associated with elevated CBF. Variability in both iron levels was linked to alterations in attention performance.*

***Clinical relevance*** *The observed associations between systemic and brain iron status and cognitive performance highlight the importance of maintaining blood–brain iron homeostasis to prevent cognitive dysfunction, particularly in individuals with iron deficiency.*

**Graphical Abstract:**

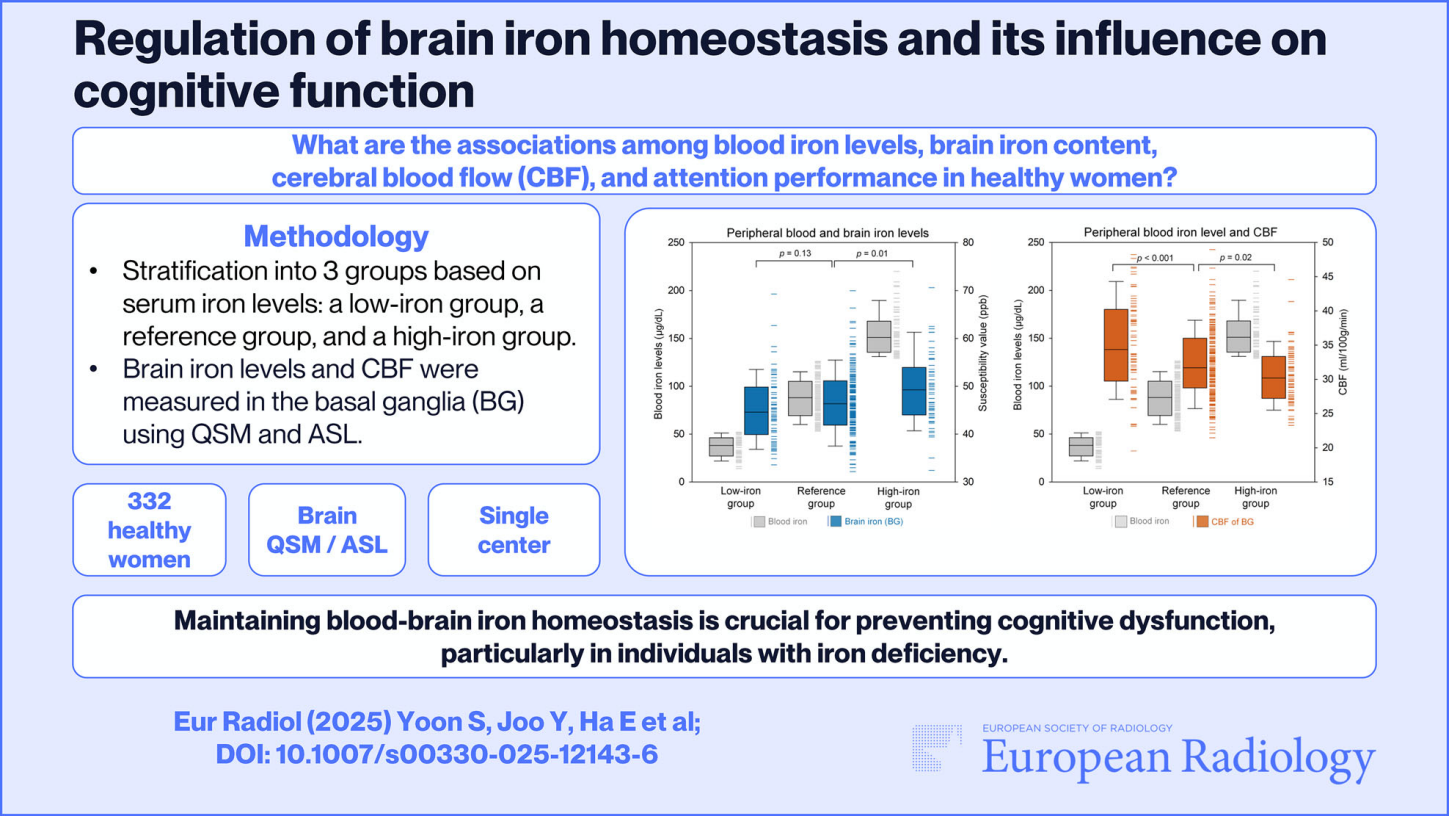

## Introduction

Iron is essential for numerous neurobiological processes, including oxygen transport, mitochondrial function, myelin synthesis, and neurotransmitter metabolism [1]. The maintenance of optimal brain iron levels is critical for supporting normal physiological function [2–4]. Disruptions in brain iron homeostasis, whether through deficiency or excess, can significantly affect cognitive function. Specifically, insufficient brain iron may hinder cognitive processes, while iron overload can exacerbate oxidative stress and promote neurodegeneration [3, 4].

Despite its importance, the regulatory mechanisms underlying brain iron homeostasis remain incompletely understood. Findings from animal studies on the effects of systemic iron deficiency and repletion on brain iron levels have been inconsistent [5–7]. Some investigations have demonstrated reduced brain iron content following dietary iron restriction [5, 6], whereas others have reported minimal changes in brain iron despite experimental iron overload [7]. Human research in this domain remains limited, highlighting the need for further investigation to clarify how systemic iron fluctuations affect brain iron regulation and cognitive outcomes.

Recent advancements in neuroimaging, particularly the development of quantitative susceptibility mapping (QSM), offer promising tools for noninvasively assessing regional brain iron content with high sensitivity and specificity [8, 9]. QSM has been validated against histological and biochemical standards, confirming its accuracy and reliability in measuring tissue iron concentrations [10, 11].

In this study, we sought to investigate the relationship between peripheral blood iron levels and brain iron content, with particular emphasis on the effects on cerebral blood flow (CBF) within the context of brain iron homeostasis. We hypothesized that CBF is essential for iron deficiency to the brain [12, 13] and aimed to investigate the adaptive mechanisms sustaining brain iron homeostasis in healthy individuals. Using a multimodal imaging approach integrating QSM and arterial spin labeling (ASL) perfusion-weighted imaging, we examined the associations among systemic iron status, brain iron levels, and CBF in healthy women. Furthermore, we explored whether systemic iron deficiency, even in the absence of anemia, may be associated with cognitive alterations, with a specific focus on attention performance.

## Materials and methods

### Participants

This prospective study, conducted between July and October 2023, enrolled healthy women of reproductive age with no history of iron-related disorders. The study protocol was approved by Ewha Womans University’s Institutional Review Board, and written informed consent was obtained from all participants. Exclusion criteria included a history of major medical or neurological conditions, psychiatric disorders, traumatic brain injury involving loss of consciousness, or contraindications to MRI.

Participants were stratified into three groups according to serum iron quintiles: a low-iron group (*n* = 67, 20.1%; mean 36.4 µg/dL, range 14.0–52.0), a reference group comprising the middle three quintiles (*n* = 200, 60.2%; mean 87.9 µg/dL, range 53.0–126.0), and a high-iron group (*n* = 65, 19.6%; mean 154.9 µg/dL, range 129.0–220.0). Low-iron group levels fell below the normal female range (50–170 µg/dL) [14], though none were clinically diagnosed with anemia at recruitment.

### Image data acquisition and processing

Multimodal MRI data, including T1-weighted, susceptibility-weighted, and pseudocontinuous ASL (pCASL) perfusion-weighted images, were acquired utilizing a 3.0 Tesla Philips dStream MR scanner system (Philips Healthcare). Acquisition parameters are detailed in Supplementary Methods.

QSM was performed on raw multi-echo gradient recalled echo (GRE) data using the SEPIA software (https://github.com/kschan0214/sepia). ASL perfusion images were processed with the FMRIB Software Library tools (FSL, http://www.fmrib.ox.ac.uk/fsl). Full descriptions of the data preprocessing pipelines are provided in Supplementary Methods.

To estimate the degree of spatial heterogeneity in each participant’s BG, we computed the within-subject voxel-wise standard deviation (SD) of the susceptibility values and likewise for CBF values within the BG ROI. This voxel-wise SD captures the variability of signal across voxels within an individual’s BG. Across the cohort, these within-subject SD values ranged from 31.1 to 87.2 ppb for susceptibility and from 5.9 to 15.4 mL/100 g/min for CBF.

### Region of interest (ROI) segmentation

The basal ganglia (BG)—caudate nucleus, putamen, and globus pallidus—were selected as the region of interest (ROI), due to their high metabolic demand and vulnerability to iron accumulation [15, 16]. Bilateral segmentation of BG structures was conducted on T1-weighted image using FSL’s FIRST software, with detailed procedures described in Supplementary Methods. The resulting segmentation masks were used to extract quantitative susceptibility or CBF values.

### Cognitive assessments

Cognitive function was assessed with a specific focus on attention, a domain known to be sensitive to iron deficiency [4, 17]. The Cambridge Neuropsychological Test Automated Battery (CANTAB®, Cambridge Cognition Ltd.) was administered to assess cognitive performance. We utilized two specific tests: Spatial Working Memory (SWM) and Reaction Time Index (RTI) [18].

The SWM test assesses the ability to retain and manipulate spatial information in working memory and top-down attentional processing [19, 20]. Participants are required to search for tokens hidden in an array of colored boxes displayed on screen. The task progressively increases in difficulty from 4 to 8 boxes, and participants must remember which boxes have been searched to avoid returning to them [18].

The RTI test comprises both simple and five-choice reaction time components to assess psychomotor speed and sustained attention [21]. In the simple reaction time phase, participants respond to a yellow dot appearing at a single, predictable location. In the five-choice reaction time phase, participants must respond to stimuli appearing unpredictably at one of five possible locations, thus incorporating elements of divided attention and decision-making [18].

While robust behavioral and neuroimaging evidence indicates functional and neurobiological overlap between spatial working memory and attention [22, 23], this overlap suggests that these theoretical constructs are very difficult to distinguish empirically. Although averaging SWM and RTI scores provides a reasonable composite measure that is related to attention performance, we acknowledge that this measure cannot be definitively separated from other cognitive domains, including fluid cognition, working memory, and executive function.

Composite attention scores were calculated by averaging the z-scores of SWM and RTI test measures. Details regarding the construction of composite scores are provided in Supplementary Methods.

### Statistical analysis

Multiple linear regression analyses were used to compare ROI susceptibility values and CBF across serum iron groups, with age included as a covariate. Group membership was coded with dummy variables, with the middle three quintiles defined as the reference category. Each regression coefficient represents the adjusted mean difference between the low-iron or high-iron group and the reference, estimated within a unified model. Since these comparisons are incorporated a priori and interpreted simultaneously as part of the regression modeling framework, no correction for multiple comparisons was applied to the regression coefficients.

Path analysis examined interrelationships among age, blood iron, ROI susceptibility, and CBF. Standardized path coefficients were estimated, and statistical inference was supported using bootstrap resampling with 5000 iterations. As the model was just-identified, overall fit indices were not evaluated [24]; therefore, interpretation focused on the statistical significance of individual direct and indirect pathways.

Robust regression models, adjusted for age, were used to investigate the effects of serum iron and brain susceptibility on CBF within each group. Interaction terms were included to assess differences in regression slopes across groups.

To explore associations between iron measures and attention performance, robust regression analyses were performed with age as a covariate. Given prior evidence suggesting a nonlinear, inverted U-shaped relationship between brain iron levels and cognitive outcomes [3, 4, 25], quadratic models were employed to capture potential curvilinear effects.

All statistical analyses were conducted using Stata SE version 16.1 (StataCorp LLC).

## Results

### Participant characteristics

Table 1 summarizes the clinical characteristics of the participants. No significant age differences were observed between the reference group and either the low-iron group (*β* = 0.07, *p* = 0.22) or the high-iron group (*β* = 0.08, *p* = 0.14).

**Table 1:** Characteristics of study participants

	Total subjects (*n* = 332)	Low-iron group (*n* = 67)	Reference group (*n* = 200)	High-iron group (*n* = 65)
Age, years	38.7 ± 5.2	39.2 ± 5.1	38.3 ± 5.3	39.4 ± 5.0
Iron, µg/dL	90.6 ± 42.3	36.4 ± 10.9	87.9 ± 20.5	154.9 ± 22.4
Ferritin, ng/mL	57.3 ± 47.4	30.4 ± 35.1	61.7 ± 48.3	71.4 ± 45.7
Hemoglobin, g/dL	12.8 ± 1.1	11.7 ± 1.4	13.0 ± 0.9	13.4 ± 0.8
Hematocrit, %	39.1 ± 2.9	36.7 ± 3.5	39.5 ± 2.5	40.3 ± 2.2
MCV, fL	89.8 ± 4.9	86.3 ± 7.0	90.4 ± 3.9	91.5 ± 3.1
MCH, pg	29.5 ± 2.2	27.5 ± 3.0	29.8 ± 1.6	30.5 ± 1.2
MCHC, g/dL	32.8 ± 1.1	31.8 ± 1.4	33.0 ± 0.9	33.3 ± 0.7
RBC, 10^6^/µL	4.36 ± 0.34	4.27 ± 0.38	4.37 ± 0.33	4.41 ± 0.30

Data are presented as mean ± standard deviation

*MCH* mean corpuscular hemoglobin, *MCHC* mean corpuscular hemoglobin concentration, *MCV* mean corpuscular volume, *RBC* red blood cell

### BG susceptibility and CBF values across the low-iron, reference, and high-iron groups

**Fig. 1:**
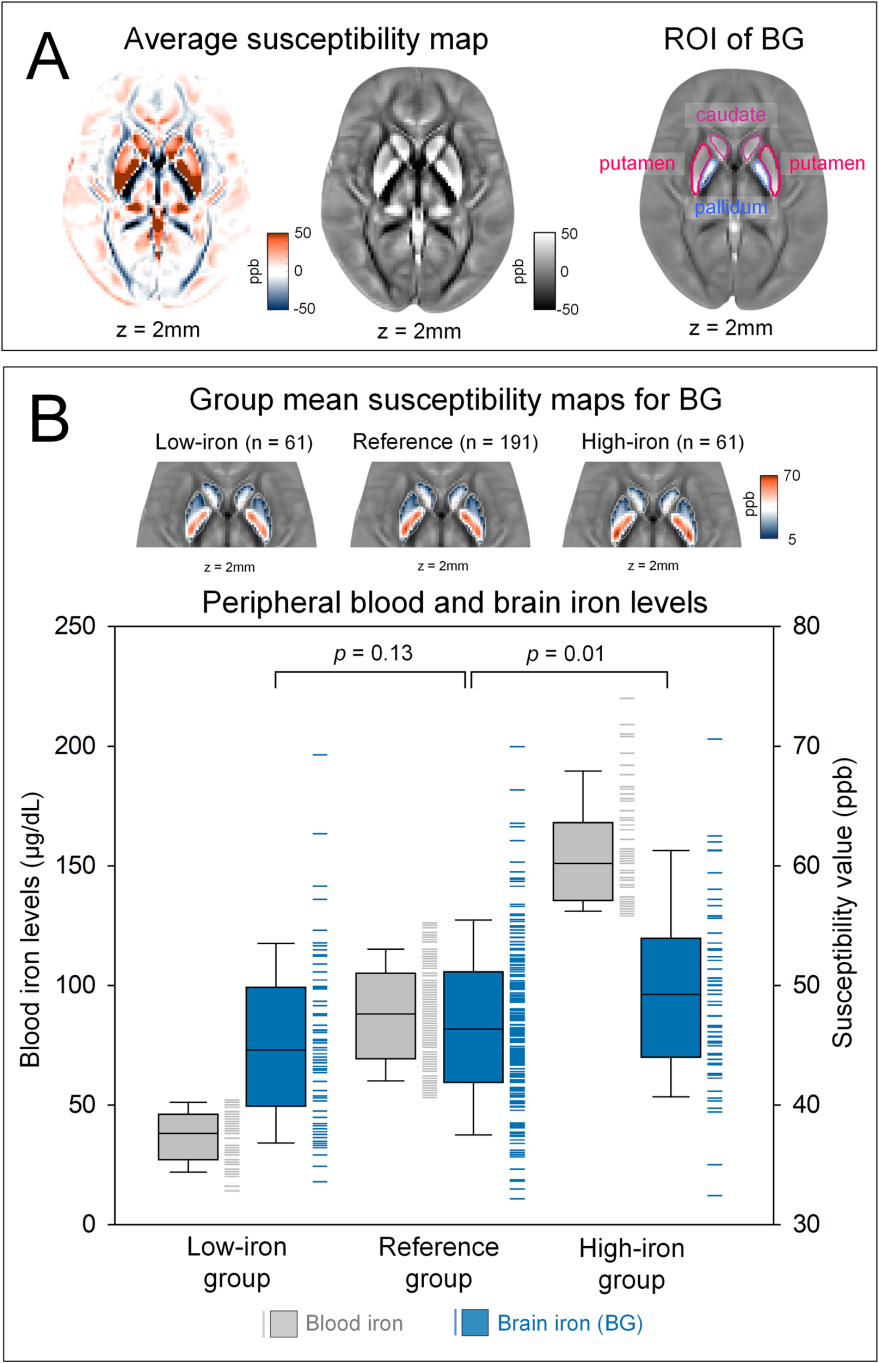
Susceptibility values across study groups. **A** Average susceptibility maps across all participants with ROIs for the BG. **B** Group mean susceptibility maps for the BG and a box-and-whisker plot illustrating blood iron levels (gray) and BG iron levels (blue). Median BG susceptibility values for each group are indicated. Multiple linear regression analysis, adjusted for age, was used to compare BG susceptibility values across groups. Participants in the combined second to fourth quintiles served as the reference group and were compared with those in the first quintile (low-iron group) and the fifth quintile (high-iron group) using dummy-coded variables. BG, basal ganglia; ROI, region of interest

Multiple linear regression analysis revealed higher BG susceptibility values in the high-iron group compared to the reference group (*β* = 0.14, *p* = 0.01), while no differences between the low-iron and reference groups (*β* = −0.08, *p* = 0.13) (Fig. 1). Regional analyses showed significant differences only in the putamen for the high-iron group (*β* = 0.12, *p* = 0.03), with no significant difference in other structures. Detailed statistics are provided in Supplementary Results.

**Fig. 2:**
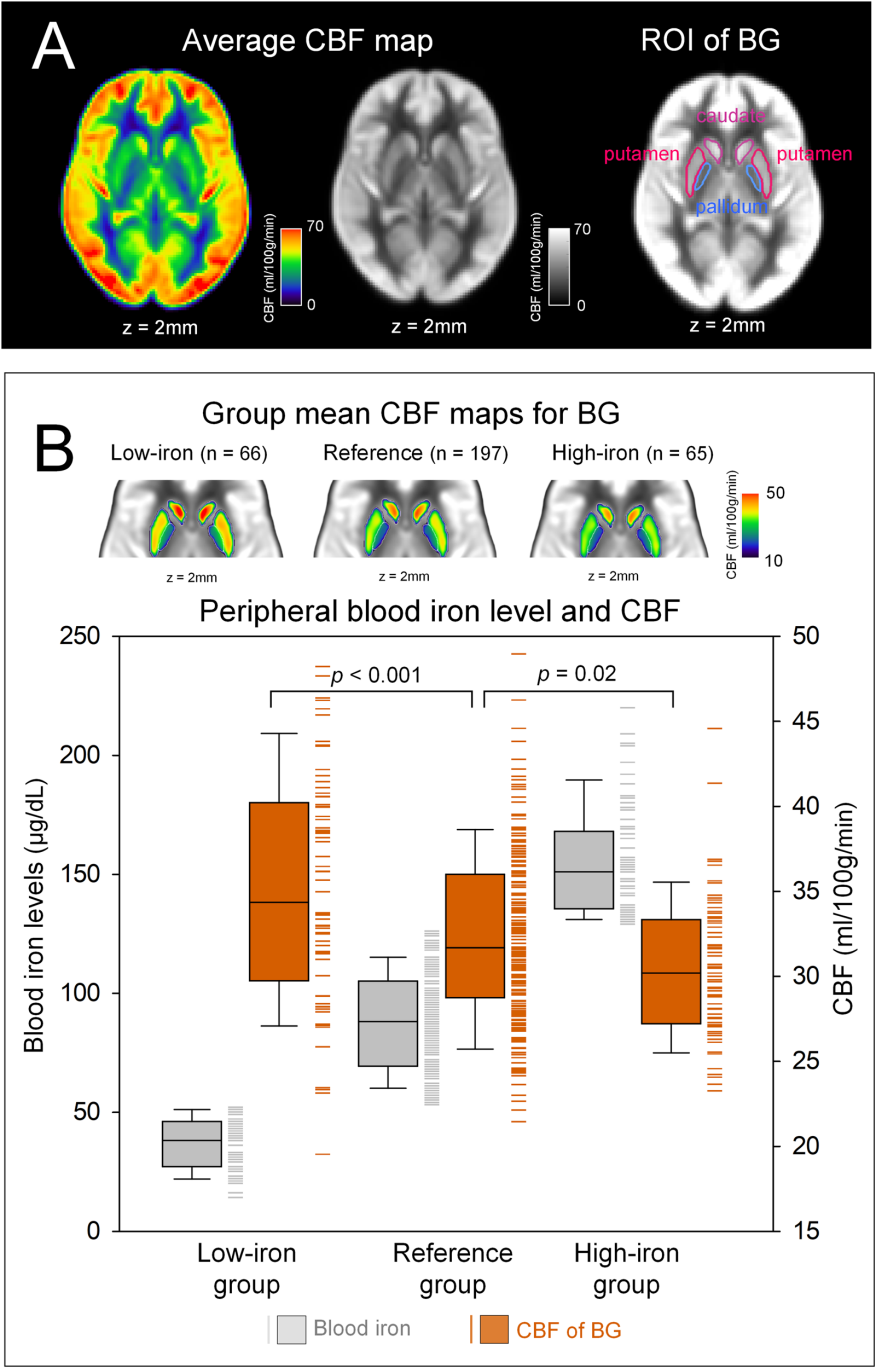
CBF values across study groups. **A** Average CBF maps across all participants with ROIs for the BG. **B** Group mean CBF maps for the BG and a box and whisker plot displaying blood iron levels (gray) and CBF values (orange). Median CBF values in the BG for each group are indicated. Multiple linear regression analysis, controlling for age, was performed to compare BG CBF across groups. Participants in the combined second to fourth quintiles served as the reference group and were compared with those in the first quintile (low-iron group) and the fifth quintile (high-iron group) using dummy-coded variables. BG, basal ganglia; CBF, cerebral blood flow; ROI, region of interest

The low-iron group showed elevated BG CBF (*β* = 0.21, *p* < 0.001), while the high-iron group showed reduced CBF, as compared to the reference group (*β* = −0.13, *p* = 0.02) (Fig. 2). These patterns were consistent across all individual BG structures. Complete statistical results are provided in Supplementary Results.

Sensitivity analyses based on ferritin quintile classification yielded comparable findings (Supplementary Results).

### Interrelationships between age, blood iron, brain iron and CBF

**Fig. 3:**
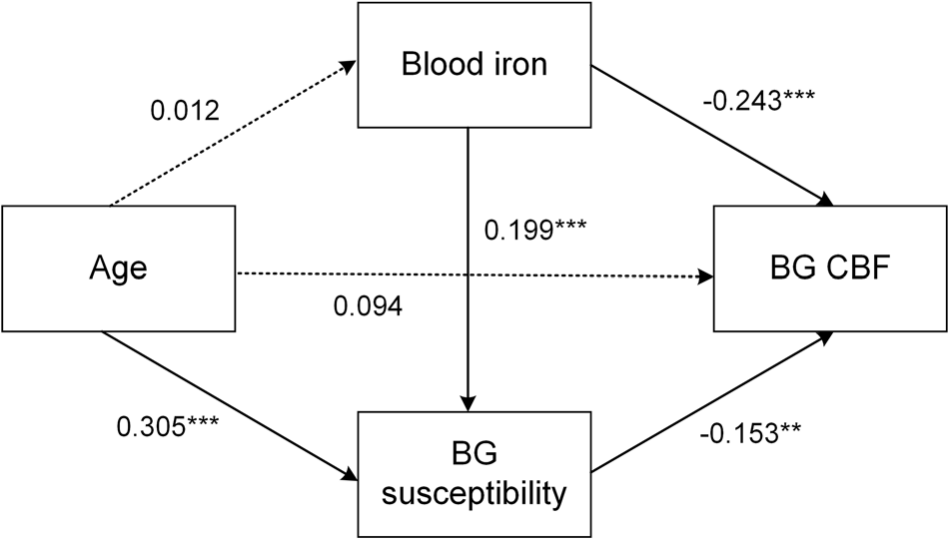
Path analysis model examining relationships among age, blood iron, BG susceptibility values, and BG CBF. The diagram illustrates standardized path coefficients representing direct associations among variables. Solid arrows indicate statistically significant paths; dashed arrows indicate non-significant associations. Age was significantly associated with BG susceptibility but not with blood iron. Blood iron levels were positively associated with BG susceptibility and negatively associated with BG CBF. BG susceptibility also showed a significant negative association with CBF. The model suggests that age influences CBF indirectly via susceptibility, while blood iron affects CBF through both direct and indirect pathways. *** *p* < 0.001, ** *p* < 0.05. BG, basal ganglia; CBF, cerebral blood flow

Path analysis revealed no significant association between age and blood iron levels (*β* = 0.01, bootstrap standard error (SE) = 0.45, *p* = 0.83), whereas advancing age was significantly associated with increased BG susceptibility (*β* = 0.31, bootstrap SE = 0.07, *p* < 0.001), consistent with previously reported age-related iron deposition [16, 26]. Lower blood iron levels were associated with decreased BG susceptibility (*β* = 0.20, bootstrap SE = 0.01, *p* < 0.001). Both reduced blood iron (*β* = −0.24, bootstrap SE = 0.01, *p* < 0.001) and decreased BG susceptibility (*β* = −0.15, bootstrap SE = 0.05, *p* = 0.01) were independently associated with elevated BG CBF, suggesting compensatory perfusion in individuals with reduced systemic and cerebral iron. Additionally, blood iron exhibited a significant indirect effect on CBF through BG susceptibility (*β* = −0.03, bootstrap SE = 0.002, *p* = 0.04), indicating that systemic iron influences brain iron levels, which in turn modulate cerebral perfusion (Fig. 3).

### Associations between blood iron, brain iron, and CBF across iron status groups

**Fig. 4:**
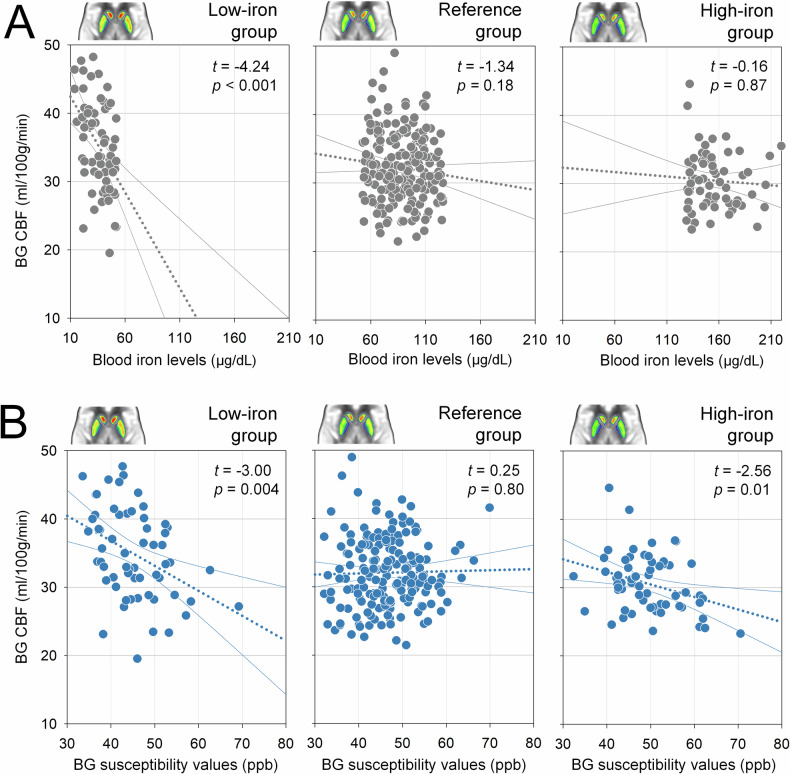
Group-specific associations of BG CBF with blood iron levels (**A**) and BG susceptibility values (**B**). Scatterplots illustrate associations for the low-iron (left), reference (middle), and high-iron (right) groups. **A** A significant negative association between blood iron levels and BG CBF was observed only in the low-iron group; no significant associations were found in the reference or high-iron groups. **B** Significant negative associations between BG susceptibility and CBF were observed in both the low-iron and high-iron groups, but not in the reference group. BG, basal ganglia; CBF, cerebral blood flow

Stratified analyses revealed distinct patterns in the relationships between iron status and BG CBF across groups (Fig. 4). In the low-iron group, both blood iron (*t* = −4.24, *p* < 0.001) and BG susceptibility (*t* = −3.00, *p* = 0.004) were negatively correlated with CBF, indicating that lower systemic and brain iron levels were associated with increased perfusion. In contrast, the reference group exhibited no significant correlations between either blood iron (*t* = −1.34, *p* = 0.18) or BG susceptibility (*t* = −0.25, *p* = 0.80) and CBF. In the high-iron group, blood iron was not associated with CBF (*t* = −0.16, *p* = 0.87), while BG susceptibility remained negatively correlated with CBF (*t* = −2.56, *p* = 0.01), suggesting that elevated brain iron, but not systemic iron, may contribute to perfusion reductions in this group.

Robust regression analyses confirmed significant differences in the regression slopes across the groups. Full results are available in Supplementary Results.

### Associations between blood iron, brain iron, and attention performance

**Fig. 5:**
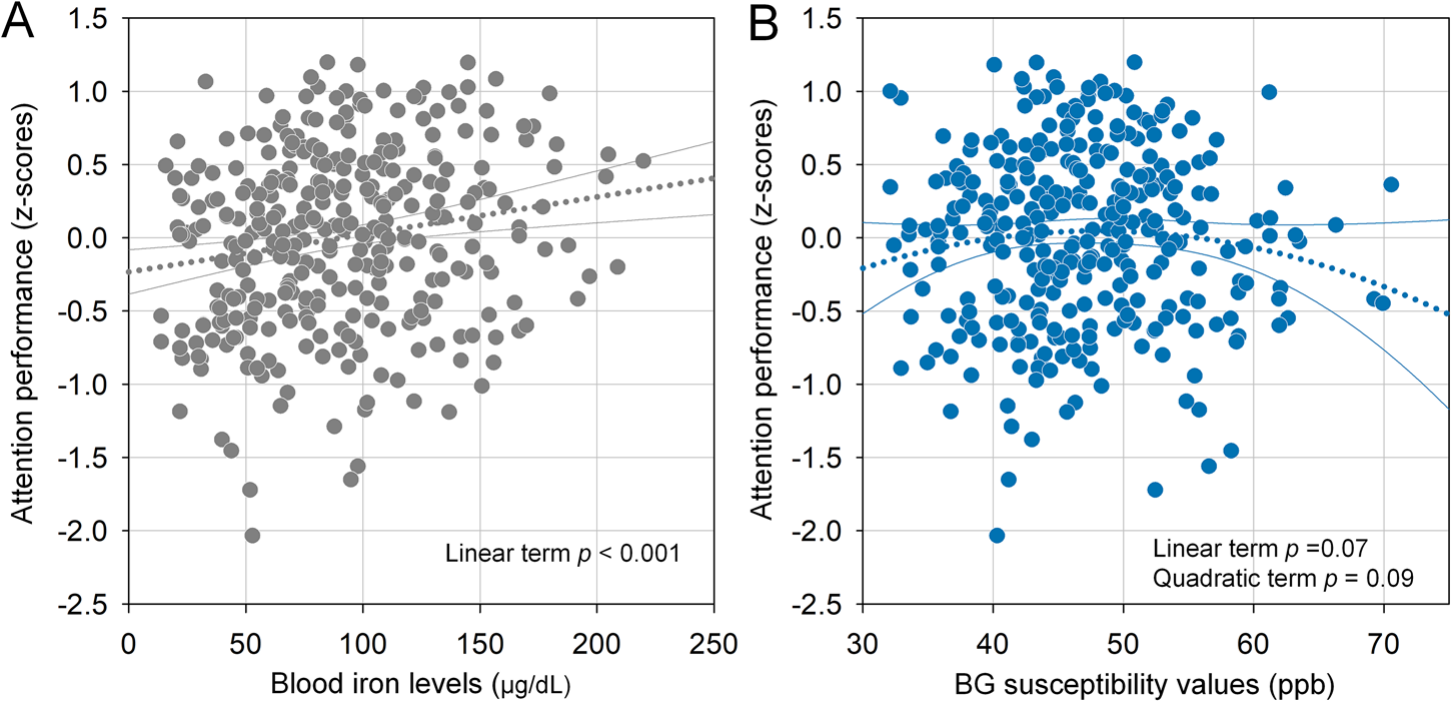
Associations between blood iron, BG susceptibility, and attention performance. Scatterplots with regression trendlines depict the relationships between **A** blood iron levels and attention performance, and **B** BG susceptibility and attention performance, adjusted for age. **A** A significant positive linear relationship was observed between blood iron levels and attention performance, indicating that lower blood iron was associated with reduced performance. **B** A nonlinear association was modeled using both linear and quadratic terms, revealing a curvilinear pattern. Although individual polynomial terms showed marginal significance, the overall model was statistically significant. BG, basal ganglia

The associations between iron indices and attention performance are shown in Fig. 5. After adjusting for age, lower blood iron levels were significantly associated with poorer attention performance (*t* = 3.21, *p* = 0.001). The relationship between BG susceptibility and attention showed a curvilinear trend: although the individual polynomial terms were only marginally significant (linear: *t* = 1.83, *p* = 0.07; quadratic: *t* = −1.71, *p* = 0.09), the overall nonlinear model reached significance (*F*_3,326_ = 3.19, *p* = 0.02), indicating that both low and high BG susceptibility values were associated with reduced attention performance.

## Discussion

This study, conducted in a relatively large cohort of reproductive-aged women, provides novel insights into the dynamic interplay between systemic iron status and cerebral iron homeostasis. Participants with lower systemic iron levels exhibited elevated CBF, compared to those with normal iron levels, suggesting the presence of an adaptive mechanism aimed at mitigating reduced iron availability. Brain iron levels demonstrated an inverse association with CBF, with both low and high iron content linked to altered perfusion patterns. Additionally, lower serum iron concentrations were associated with diminished attention performance, while brain iron content exhibited a curvilinear relationship with attention outcomes, indicating the existence of an optimal range for cognitive function. To our knowledge, this is the first study to investigate the association between CBF and fluctuations in both systemic and brain iron levels under conditions of subclinical iron deficiency. These findings contribute to a more comprehensive understanding of brain iron regulation and may inform preventive strategies targeting the cognitive consequences of early-stage iron deficiency.

The observed elevation in CBF among participants in the low-iron group is indicative of an adaptive cerebrovascular response designed to maintain adequate oxygen delivery in the context of reduced systemic oxygen-carrying capacity. Given iron’s critical role in oxygen transport and cellular respiration, along with the brain’s disproportionately high oxygen demands, preserving iron delivery to the brain is essential for sustaining optimal neuronal function [12, 13]. The marked increase in CBF in the low-iron group likely reflects a physiological adaptation to enhance cerebral iron delivery under systemic iron deficiency. This interpretation is further supported by the significant inverse correlation between systemic iron levels and CBF within this group. These findings are consistent with prior studies reporting elevated CBF in anemic hemodialysis patients [13], reinforcing the concept that increased CBF may serve as an adaptive mechanism to support cerebral iron homeostasis.

Importantly, despite systemic iron depletion, no significant differences in BG susceptibility were detected between the low-iron and reference groups. This suggests that decreased systemic iron does not necessarily result in proportional reductions in brain iron stores. Although path analysis revealed a significant direct association between systemic and brain iron levels, the elevated CBF observed in the low-iron group may help preserve cerebral iron content. This finding aligns with previous research suggesting that brain iron levels remain relatively stable despite fluctuations in systemic iron availability, due to intrinsic homeostatic mechanisms [27, 28].

In contrast, participants in the high-iron group exhibited significantly elevated BG susceptibility compared to the reference group, consistent with the hypothesis that increased systemic iron levels can lead to excessive brain iron deposition. Path analysis further indicated that elevated systemic iron contributes to enhanced iron accumulation in the BG, which in turn is associated with reduced CBF. This pattern supports the notion that excessive brain iron may impair cerebrovascular function, potentially through mechanisms involving oxidative stress or endothelial dysfunction [29, 30].

Regional analysis revealed that the observed group differences in susceptibility within the high-iron group were predominantly driven by the putamen, with no significant differences in the caudate or globus pallidus. This regional specificity suggests differential vulnerability and regulation of iron accumulation within BG substructures. The putamen’s heightened susceptibility to iron deposition may reflect its specialized role in motor control and procedural learning [31], as well as its documented involvement in motor impairment across various neurodegenerative conditions [32]. These observations underscore the need for future studies to investigate region-specific patterns of brain iron accumulation in the context of systemic iron variation.

Path analysis demonstrated a positive association between blood iron levels and BG susceptibility values, confirming that systemic iron availability directly influences cerebral iron content. While advancing age remains the predominant factor contributing to brain iron accumulation, systemic iron status also plays a measurable role in determining overall cerebral iron load. These findings suggest that limiting systemic iron excess may help attenuate age-related increases in brain iron, consistent with observations from dietary intervention studies [33].

Path analysis further revealed that both lower blood iron levels and reduced BG susceptibility were associated with elevated BG CBF. Notably, blood iron exhibited a significant indirect effect on CBF via brain iron content, establishing a mechanistic pathway in which systemic iron influences cerebral perfusion through its impact on local iron deposition. This finding highlights the active regulatory role of brain iron in cerebrovascular homeostasis, extending beyond its function as a passive biomarker. Accordingly, maintaining optimal brain iron levels—regardless of systemic status—appears critical for supporting healthy cerebrovascular function. Dysregulation of cerebral iron, whether through excess or deficiency, may impair CBF and thereby compromise cerebral metabolic integrity.

Consistent with previous reports [16, 26], increasing age was significantly associated with higher BG susceptibility values, with path analysis confirming age as the strong determinant of cerebral iron accumulation. In contrast, no significant age-related increase in blood iron levels was observed in our cohort. This dissociation suggests that age-related brain iron accumulation may occur even in individuals with stable or normal systemic iron levels, reflecting the brain’s intrinsic vulnerability to iron deposition in specific regions such as the BG during aging [16, 26].

To further clarify the relationships among systemic iron, brain iron, and CBF, we conducted stratified analyses by iron status group. In the low-iron group, both serum and brain iron levels were positively associated with increased CBF. In contrast, in the high-iron group, elevated BG susceptibility was linked to reduced CBF, while serum iron levels were not significantly associated with perfusion. These findings suggest that once iron crosses the blood–brain barrier and accumulates in brain tissue, it may exert local effects on vascular regulation independent of circulating iron levels [13]. This pattern supports a context-dependent cerebral iron response under iron-deficient conditions; elevated CBF may reflect a compensatory mechanism to support oxygen delivery or metabolic efficiency [13]; under iron-overload conditions, excess cerebral iron may impair perfusion through mechanisms involving oxidative stress and neuroinflammation, resulting in localized hypoperfusion irrespective of systemic iron status [3, 29, 30]. Collectively, these results underscore the importance of brain-specific iron homeostasis in preserving cerebrovascular function. Nonetheless, longitudinal studies examining the temporal evolution of cerebral iron accumulation and its impact on CBF are warranted to validate this proposed framework.

Within the established reference range for serum iron levels in women (50–170 µg/dL) [14], our study participants exhibited a wide spectrum of iron status. The high-iron group presented levels near the upper physiological limit (mean 154.9 µg/dL, range 129–220 µg/dL), whereas the low-iron group displayed sub-normal levels (mean 30.4 µg/dL, range 14.0–52.0 µg/dL), despite the absence of clinically diagnosed anemia. This distribution enabled the investigation of cognitive consequences associated with iron status across and below the normative range. Our findings revealed a significant association between reduced serum iron and impaired attention performance. This observation is consistent with extensive evidence indicating that non-anemic iron deficiency—as represented by the low-iron group—negatively impacts cognitive and behavioral function, particularly in populations at elevated risk such as reproductive-aged women [34]. These effects are believed to be mediated by disruptions in brain activity and neurotransmitter metabolism [35, 36], emphasizing the importance of adequate iron status for optimal neurological performance, even in the absence of overt anemia.

Beyond the linear association between systemic iron deficiency and cognitive impairment, our analysis revealed a curvilinear relationship between brain iron content and attention performance. Specifically, both low and high BG susceptibility values were associated with diminished performance, suggesting an inverted U-shaped association. This pattern reflects iron’s dual role in neural function: insufficient iron may impair neurotransmitter synthesis and oxygen transport [4], while excess iron can induce oxidative stress and neuroinflammation [2, 3]. These results are in line with prior findings [37, 38] and underscore the need to maintain brain iron within an optimal range to support cognitive health.

Although this study focused on a composite measure related to attention—a cognitive domain particularly sensitive to iron deficiency [4, 17]—it is important to acknowledge that our averaged scores from the SWM and RTI tasks do not constitute a definitive measure of attention alone. Given the substantial functional and neurobiological overlap between attention, spatial working memory, and related constructs [22, 23], it is empirically challenging to distinguish this composite measure from other domains such as fluid cognition, working memory, and executive function. Brain iron accumulation has also been linked to age-related declines in these broader domains [2, 39, 40], and our findings may reflect influences on this interconnected cognitive network rather than attention in isolation. Consequently, further investigation into the effects of cerebral iron accumulation on a broader spectrum of cognitive functions, using more granular and multifaceted assessments, is warranted to gain a more comprehensive understanding of its role in cognitive aging and dysfunction.

In this study, the BG were selected as the ROI due to their high iron content. Brain iron is primarily stored in ferritin within microglia and astrocytes [41]. and these subcortical nuclei are known for their high metabolic demand and vulnerability to both iron deficiency and overload [15, 16, 42]. Future studies should extend this work by examining the impact of systemic iron on iron regulation in additional brain regions beyond the BG.

We selected serum iron as the primary index of iron status to capture dynamic changes at the time of CBF measurement. While serum ferritin reflects long-term iron stores as a storage protein [43], it is less responsive to acute fluctuations. Despite this methodological distinction, our sensitivity analyses using ferritin-based groupings produced similar results, supporting the robustness of our primary findings. Nevertheless, future longitudinal research is needed to delineate the interplay between acute and chronic iron status in regulating brain iron and cerebral perfusion.

Several limitations should be considered when interpreting these results. The study cohort consisted exclusively of reproductive-aged women, given their heightened vulnerability to iron deficiency [44, 45]; thus, the generalizability of the findings to men and postmenopausal women may be limited. Previous studies have reported mixed findings regarding sex differences in brain iron levels [26, 46–51], highlighting the need for future research involving more diverse populations to validate and extend these results.

A key methodological limitation is the exclusion of cognitive outcomes from the path analysis model. Although age, blood iron, brain iron, and CBF were modeled simultaneously to examine their interrelationships, cognitive performance was analyzed separately via regression due to the nonlinear (inverted U-shaped) relationship between brain iron and attention performance, which violates the linearity assumptions of traditional path analysis [24, 52]. This quadratic pattern—where both low and high brain iron levels were linked to diminished attention—aligns with evidence that iron deficiency and excess impair cognition [3, 4]. Incorporating nonlinearity into a linear model risks misspecification and misleading effect estimates. Consequently, our cognitive findings remain correlational, and we cannot determine whether blood iron’s association with attention is mediated by CBF or direct. Future studies could address this by using nonlinear extensions or subgroup analyses (e.g., in groups with low blood iron/reduced brain susceptibility or high brain susceptibility) to model causal pathways, potentially with age as a predictor and iron/CBF as mediators, for deeper insights into cognitive mechanisms.

Additionally, a limitation of our study that warrants discussion is the interpretation of variance within our quantitative MRI measures and the inherent precision of these techniques. While this variability largely stems from biological heterogeneity within the BG (e.g., subregional gradients in susceptibility across caudate, putamen, and globus pallidus), it may also include contributions from measurement noise, reconstruction artifacts, or partial volume effects. This substantial within-ROI variability means that even statistically significant group differences have modest effect sizes relative to the background heterogeneity, potentially limiting their practical significance, especially at the individual level. Caution is therefore warranted in interpreting small differences. Future studies should incorporate repeated scans to formally assess measurement reliability (e.g., via intraclass correlation coefficients) and explore subregion-specific variations using higher-resolution imaging or advanced segmentation techniques.

Finally, although other paramagnetic or diamagnetic trace elements and changes in myelin content could theoretically influence magnetic susceptibility values, their contribution is likely minimal relative to that of iron, which is the predominant source of susceptibility contrast in brain tissue [53].

In conclusion, this study demonstrates that both systemic and brain iron levels play a critical role in regulating cerebral blood flow when systemic iron deviates from physiological norms. Iron deficiency, even in the absence of anemia, was associated with impaired cognitive performance, specifically in the domain of attention. Furthermore, the data suggest that maintaining optimal brain iron levels is essential for preserving cognitive function. Collectively, these findings underscore the importance of blood–brain iron homeostasis in supporting cerebral perfusion and cognitive performance, particularly in individuals vulnerable to iron deficiency.

## Supplementary information

ELECTRONIC SUPPLEMENTARY MATERIAL

## Abbreviations

ASL: Arterial spin labeling
BG: Basal ganglia
CBF: Cerebral blood flow
FSL: FMRIB Software Library
MRI: Magnetic resonance imaging
QSM: Quantitative susceptibility mapping
ROI: Region of interest
RTI: Reaction time
SE: Standard error
SWM: Spatial Working Memory
