# Supplementary material for: Regulation of brain iron homeostasis and its influence on cognitive function
Source: Eur Radiol. 2026 Jan 22;36(6):5140–52. doi: 10.1007/s00330-025-12143-6 (PMC13212747; doi:10.1007/s00330-025-12143-6)
Supplement: Supplementary file 1 — ELECTRONIC SUPPLEMENTARY MATERIAL [file 330_2025_12143_MOESM1_ESM.pdf]

Regulation of brain iron homeostasis and its influence on cognitive function

ELECTRONIC SUPPLEMENTARY MATERIAL

SUPPLEMENTARY METHODS

Image data acquisition

Multimodal MRI data were collected utilizing a dStream 3.0 Tesla Philips MR scanner system (Philips Healthcare, Best, The Netherlands) equipped with a 32-channel head coil. High-resolution, three-dimensional T1-weighted images were obtained using a turbo field echo sequence. The acquisition parameters included: repetition time (TR)=7.4 ms, echo time (TE)=3.4 ms, flip angle=8º, and field of view (FOV)=224 (AP) × 224 (FH) × 180 (RL) mm^3 with reconstruction voxel size=1×1×1 mm^3, compressed sensing-sensitivity encoding (CS-SENSE) factor=4, and scan duration=3 minutes.

Susceptibility-weighted MRI data were acquired using a three-dimensional multi-echo gradient-recalled echo (GRE) sequence with the following parameters: TR=63 ms, 10 echoes acquired at TE1/TE spacing/TE10=5.6/6/59.6 ms, flip angle=17º, FOV=220 (AP) × 202 (RL) × 150 (FH) mm^3 with reconstruction voxel size=0.6×0.6×1 mm^3, CS-SENSE factor=6, and scan duration=5 minutes.

Pseudocontinuous arterial spin labeling (pCASL) perfusion-weighted MRI data were acquired using a three-dimensional single-shot gradient and spin echo (3D-GRASE) sequence. The acquisition parameters were: TR=3,772 ms, TE=12 ms, FOV=220 (AP)X220 (RL)X132 (FH) mm^3 with reconstruction voxel size=2.75X2.75X6 mm^3, labeling duration=1,800 ms, and post labeling delay=1,600 ms. Additionally, an echo-planar imaging proton density (M0) image was obtained to estimate the equilibrium magnetization of arterial blood. This image was acquired without labeling or background suppression, using the same acquisition parameters as the pCASL scan, except the TR was adjusted.

Region of interest (ROI) segmentation

FIRST utilizes a Bayesian model-based method, incorporating prior anatomical knowledge to ensure precise and reliable segmentation. Initially, the T1-weighted image was aligned linearly to the MNI152 standard space, followed by boundary adjustment to enhance the accuracy of subcortical segmentation. The segmented images were subsequently reverted to the original T1-weighted image's native space for thorough quality assessment and manual adjustments were made as necessary. To extract susceptibility or CBF values, the first-echo magnitude images or calibrated CBF images of each subject were coregistered to their respective T1-weighted images through affine transformations. Inverse transformation matrices were applied to adjust the ROI masks for each basal ganglia (BG) structure from T1 to QSM or ASL space.

QSM processing

The main steps of the preprocessing included combining echoes, phase unwrapping, removing background fields, and performing dipole inversion to reconstruct susceptibility maps [1].

Specifically, an initial brain mask was created for each participant using FSL BET [2] on the first-echo magnitude image and further refined by utilizing the R2* map to exclude high R2* voxels at the brain edge. This mask effectively confined QSM processing to brain tissue, thereby minimizing susceptibility measurement distortions from extracerebral elements. Echo phases were combined using optimal weights [3], and phase unwrapping was performed using the Laplacian-based method in SEPIA [4] to ensure continuous phase data. Subsequently, background field removal was applied to the unwrapped phase images using the V-SHARP methods [5] with spherical mean value filtering sizes ranging from 12 mm to 1 mm. This critical step isolated the local field induced by tissue susceptibility from other magnetic field sources. The local field map was then employed to compute the susceptibility map through dipole inversion using the iLSQR (iterative least squares with QR decomposition) algorithm [5]. The

resulting susceptibility maps, which reflect the distribution and concentration of magnetic materials such as iron within the brain, underwent visual inspection for quality control.

ASL imaging processing

The preprocessing steps included skull stripping, motion correction, pairwise subtraction of control and labeled images, and averaging to produce mean perfusion-weighted images. CBF was quantified at the voxel level by estimating the equilibrium magnetization of arterial blood using a saturation inversion recovery approach, alongside mean cerebrospinal fluid magnetization (M0) images [6]. A variational Bayesian method, implemented in the Bayesian Inference for Arterial Spin Labeling (BASIL) tool (http://fsl.fmrib.ox.ac.uk/fsl/fslwiki/BASIL) [7], was employed for calibrating perfusion parameters and evaluating model fit. This approach produced calibrated images that were subsequently converted into absolute CBF maps (ml/100g/minute) utilizing a single-compartment model [6]. Images underwent visual inspection to identify and exclude those of poor-quality.

Cognitive assessments

Individual raw scores for each outcome measure including the total number of between errors and strategy scores from the SWM task and reaction time and movement time for both simple and five-choice reaction time trials in the RTI task were converted to z-scores, and scores were adjusted as necessary to ensure that higher z-scores consistently indicated superior performance. Composite scores for assessing attention performance were calculated by averaging the z-scores of the outcome measures from both the SWM and RTI tasks. One participant from the reference group was unable to complete the SWM task, resulting in a final sample size of 331 for the cognitive assessments.

SUPPLEMENTARY RESULTS

Susceptibility and CBF values in each BG structure across the low-iron, reference, and high-iron groups

Group-wise comparisons of susceptibility values were conducted for each constituent structure within the basal ganglia ROI. No significant differences were observed between the low-iron and reference groups in the caudate (β=−0.07, p=0.21), putamen (β=−0.03, p=0.53), or globus pallidus (β=−0.09, p=0.11). However, the high-iron group exhibited significantly greater susceptibility in the putamen (β=0.12, p=0.03) compared to the reference group, whereas no significant differences were noted in the caudate (β=0.08, p=0.15) or globus pallidus (β=0.08, p=0.16).

CBF was significantly elevated in the low-iron group across all three structures: caudate (β=0.20, p<0.001), putamen (β=0.19, p=0.001), and globus pallidus (β=0.23, p<0.001), relative to the reference group. In contrast, the high-iron group demonstrated significantly reduced CBF in the caudate (β=−0.14, p=0.02) and putamen (β=−0.13, p=0.02), while the reduction in globus pallidus CBF did not reach statistical significance (β=−0.09, p=0.10).

Susceptibility and CBF values across ferritin quintile groups

As a sensitivity analysis, group comparisons of BG susceptibility and CBF values were repeated based on ferritin quintile classification.

Participants were divided into three groups: low-ferritin (lowest quintile, n=70, mean ferritin=10.5 ng/mL), reference (intermediate three quintiles, n=197, mean ferritin=48.8 ng/mL), and high-ferritin (highest quintile, n=65, mean ferritin=133.4 ng/mL). The observed patterns were consistent with those from the iron-quintile analysis. Specifically, there was no significant difference in BG susceptibility between the low-ferritin and reference groups (β=−0.08, p=0.17), while the high-ferritin group demonstrated significantly higher susceptibility values (β=0.14,

p=0.01). For CBF, the low-ferritin group exhibited significantly higher values compared to the reference group (β=0.21, p<0.001), whereas the high-ferritin group did not differ significantly (β=−0.08, p=0.16).

Differences in regression slopes for blood or brain iron-CBF associations across groups

Robust regression analyses were conducted to assess interaction effects between group status and the relationship of blood or brain iron with CBF. The association between blood iron levels and BG CBF differed significantly between the reference and low-iron groups (t=−4.26, p<0.001). A similar interaction effect was observed for BG susceptibility values between these groups (t=−3.52, p=0.001). When comparing the reference and high-iron groups, a significant interaction was noted for BG susceptibility (t=−2.30, p=0.02), while the interaction for blood iron levels was not statistically significant (t=0.61, p=0.54).

REFERENCES FOR THE SUPPLEMENTARY MATERIAL

1. QSM Consensus Organization Committee, Bilgic B, Costagli M et al (2024) Recommended implementation of quantitative susceptibility mapping for clinical research in the brain: a consensus of the ISMRM electro-magnetic tissue properties study group. Magn Reson Med 91:1834–1862
2. Smith SM (2002) Fast robust automated brain extraction. Hum Brain Mapp 17:143–155
3. Robinson SD, Bredies K, Khabipova D, Dymerska B, Marques JP, Schweser F (2017) An illustrated comparison of processing methods for MR phase imaging and QSM: combining array coil signals and phase unwrapping. NMR Biomed 30:e3601
4. Schofield MA, Zhu Y (2003) Fast phase unwrapping algorithm for interferometric applications. Opt Lett 28:1194–1196
5. Li W, Wu B, Liu C (2011) Quantitative susceptibility mapping of human brain reflects spatial variation in tissue composition. Neuroimage 55:1645–1656
6. Alsop DC, Detre JA, Golay X et al (2015) Recommended implementation of arterial spin-labeled perfusion MRI for clinical applications: a consensus of the ISMRM perfusion study group and the European consortium for ASL in dementia. Magn Reson Med 73:102–116
7. Chappell MA, MacIntosh BJ, Donahue MJ, Günther M, Jezzard P, Woolrich MW (2010) Separation of macrovascular signal in multi-inversion time arterial spin labelling MRI. Magn Reson Med 63:1357–1365
